# Establishment of normal reference values of NT-proBNP and its application in diagnosing acute heart failure in children with severe hand food and mouth disease: Erratum

**DOI:** 10.1097/MD.0000000000019688

**Published:** 2020-03-20

In the article, “Establishment of normal reference values of NT-proBNP and its application in diagnosing acute heart failure in children with severe hand food and mouth disease”,^[1]^ which appears in Volume 97, Issue 36 of *Medicine*¸ the word foot was misspelled as food in the title and the title should be “Establishment of normal reference values of NT-proBNP and its application in diagnosing acute heart failure in children with severe hand foot and mouth disease”.
